# Hemodynamically significant anemia as an indication of transfusion in preterm infants

**DOI:** 10.1186/s13052-025-01978-w

**Published:** 2025-05-16

**Authors:** Marwa Mohamed Farag, Mohamed Alaa Eldin Hassan Thabet, Ahmed Adel Hassan El Beheiry, Bahaa Salah-El Din Hammad, Mohammed Attia Khalifa, Amany Abdel Hamed Elsebaee

**Affiliations:** 1 Assistant Professor of Pediatrics and Neonatology, Pediatric Department, Alexandria University Hospital, Alexandria, Egypt; 2 Pediatric Department, Professor of Pediatrics and Neonatology, Alexandria University Hospital, Alexandria, Egypt; 3 Assistant Professor of Radiodiagnosis and Intervention, Radiology Department, Alexandria University, Alexandria, Egypt; 4 Consultant of Pediatrics and Neonatology Alexandria University Hospital, Pediatric Department, Alexandria University, Alexandria, Egypt; 5 Specialist of Pediatrics and Neonatology, Alexandria University Hospital, Pediatric department, Alexandria, Egypt; 6 Specialist of Pediatrics and Neonatology Alexandria University Hospital, Pediatric Department, Alexandria University, Alexandria, Egypt

**Keywords:** Left ventricular output, Functional echocardiography, RBCs transfusion, Anemia of prematurity, Doppler in neonate

## Abstract

**Background:**

To transfuse or not to transfuse premature infants” is still one of debatable issues in neonatal care that has not been completely solved. Recognizing hemodynamically significant (HS) anemia might be part of the solution. Our purposes were to investigate the hemodynamic effects of late onset anemia and red blood cells (RBCs) transfusion on premature neonates with gestational age 28–32 weeks and to tailor transfusion needs based on hemodynamic variables and Hb/HCT level.

**Methods:**

In the current study, 36 anemic preterm infants with a gestational age of 28–32 weeks and a postnatal age of 3–9 weeks, all having a hematocrit (HCT) level below 30% and being candidates for red blood cell (RBC) transfusions, were compared to 36 non-anemic infants with HCT levels of 30% or higher in terms of hemodynamic parameters during their initial scans. Each anemic infant underwent a second scan 24 h after receiving RBC transfusions. The hemodynamic parameters assessed included left ventricular cardiac output (LVO) and the flow velocities in the renal (RA), anterior cerebral (ACA), and celiac (CA) arteries, measured using functional echocardiography and Doppler imaging. The hemodynamic changes were related to the presence of clinical signs in the anemic infants.

**Results:**

LVO, stroke volume (SV), heart rate (HR), and ACA-peak systolic velocity (PSV) were significantly increased in anemia of prematurity and significantly decreased after RBCs transfusions. With an area under the ROC curve of 0.862, LVO displayed the highest diagnostic performance for HS-anemia of prematurity.

**Conclusions:**

LVO, SV, HR, ACA-PSV, hemodynamic parameters can be used for diagnosing HS-anemia and can provide objective criteria for identifying patients in need of RBCs-transfusions. They also help in monitoring response of RBCs-transfusion in anemic preterm infants. Those cut off measures require validation by future studies.

**Supplementary Information:**

The online version contains supplementary material available at 10.1186/s13052-025-01978-w.

This work was registered on 2022-08-09 in clinicaltrial.gov with ID no: NCT05496400 and URL: https://clinicaltrials.gov/study/NCT05496400.

## Background

Despite more than 90% of extremely low-birth-weight infants receive at least one RBC-transfusions during their NICU stay [1], when to transfuse preterm infant is still a matter of debate.

On the one hand, transfusions in newborns expose them to potential transfusion-related risks. The underdeveloped cardiovascular system of premature infants and the potential for immunological incompatibility due to the prenatal passive transfer of maternal antibodies may increase these risks [2]. Furthermore, this age group faces specific transfusion risks, which may arise from ischemia-reperfusion injury or oxidative damage, leading to conditions such as transfusion-associated necrotizing enterocolitis (NEC), bronchopulmonary dysplasia (BPD), and retinopathy of prematurity (ROP) [3–7]. 

On the other hand, decreased oxygen delivery to tissues, anemia related manifestations, and possible impaired neurocognitive-development might direct the neonatologists to transfuse the premature neonates [8–10]. 

A systematic review of 6 RCTs involving 3483 participants found no differences between high and low transfusion thresholds concerning short- and long-term outcomes [11]. However, they concentrated on Hb/Hct levels, postnatal age, and respiratory support. Investigating other aspects of anemia, such as hemodynamic changes that represent the energy cost to compensate for anemia, might assist clinicians in resolving the clinical dilemma regarding real-time indications for PRBC transfusions (packed red blood cells) in neonates while the patient is still in the NICU.

In the current study, we aim to determine if hemodynamic compensatory mechanisms and/or end-organ blood flow can aid in resolving this debate and identify anemic neonates who will benefit from RBC transfusion. Additionally, we seek to establish a concept of hemodynamically significant anemia (HS-anemia) in the decision-making process for PRBC transfusion. The primary objective of this study was to examine the hemodynamic changes associated with anemia of prematurity (AOP) in neonates with a gestational age of 28–32 weeks and the impact of RBC transfusion on those hemodynamics.

## Methods

A cross sectional study was conducted in the NICU of Alexandria University maternity Hospital in Egypt, a major tertiary center serving four governments, from June 2021 to February 2022. The study aimed to assess the hemodynamic changes provoked by AOP and RBC transfusions in stable preterm infants with a gestational age of 28–32 weeks and a postnatal age of 3–9 weeks who were not receiving respiratory support. Thirty-six non-anemic inpatient premature infants with hematocrit ≥ 30% (group I) were compared with 36 pre-transfusion anemic inpatient premature infants (group II) who were candidates for PRBCs transfusions. The anemic premature infants were assessed shortly before and 22–26 h after RBCs-transfusions in term of clinical, hematological, and hemodynamic parameters. Then, anemic patients were further sub-grouped into symptomatic and asymptomatic groups. *Patients in both groups were matched based on gestational age*,* post-natal age(weeks)*,* and gender*. Patients with hemolytic disease of the newborns, shock, sepsis and congenital anomalies including ductus arteriosus were excluded from the study.

### Definition of anemia and PRBCs-transfusion

Despite, neonatologists cannot agree on the cutoff point for Hb/HCT-to define preterm anemia, in the present study, we utilized HCT 30%, which is nearly equal to Hb 10 g/dl as it represents the lower threshold of well-tolerated anemia in a stable neonate [12]. Based on Kasat-et-al guidelines of PRBCs-transfusion in neonates [7], anemic patients received 15 ml /kg PRBCs transfusion over 4 h when they had HCT < 30% along with symptoms of anemia (symptomatic anemic group *n* = 24). We examined 24 symptomatic-anemic-infants; nineteen-infants were tachycardic > 160 beat/min, nine-infants had desaturation, one-infant had apnea and one-infant failed to thrive (≥ 1 symptom-per–patient). When there were no symptoms, transfusion was only considered when HCT value was ≤ 21% with absolute reticulocyte count < 100,000/µL or < 5% (asymptomatic anemic group *n* = 12) [9]. The 5th and 95th centiles for absolute reticulocyte counts at corresponding postnatal age range between 30,000/ µL and 200,000/ µL [13]. 

Clinical evaluations include heart rate, arterial blood pressure (systolic, diastolic and mean-BP), oxygen saturation and weight were recorded on admission and at time of scans. Patients of both groups at time of scans were off oxygen and off inotropes.

Laboratory evaluations include complete blood picture including all blood indices were recorded on admission and at time of scans.

Imaging echocardiographic and Doppler studies were performed by single operator who was not blind to patients’ groups. The imaging was done using machine: model GE Vivid iq premium, WUXI, China (M, 2D, color Doppler modes, and pulsed wave Doppler). During scans, patients were sleeping or quietly resting, in a supine position, and on a flat surface. The following parameters were measured: [14, 15]

1. Left ventricular output (LVO).

Aortic root diameter was assessed from the parasternal long-axis view at the valve hinge points at end systole. Velocity time integral (VTI) was measured from an optimized apical five chamber view by placing the pulsed-wave Doppler gate at the level of the aortic valve and averaged over 3 consecutive cycles. Stroke volume (SV) is a product of VTI and the cross-sectional area (CSA) of aortic root. The heart rate was measured from the peak-to-peak intervals of the Doppler velocity time signals.

$$\begin{aligned}\text{LVO}&=\text{SV}\times\text{HR}\\&\quad=\text{VTI}\:(\text{cm})\times\text{CSA}\:(\text{cm}^{2})\\&\quad\times\text{Heart Rate}\:(\text{Beat}/\text{min})\end{aligned}$$

LVO was divided by the weight of the preterm infant to obtain LVO in ml/kg/min.

2. Anterior cerebral artery (ACA) flow velocities measurement by transcranial Doppler-ultrasonography: by placing the transducer in the midsagittal plane via the anterior fontanelle.
3. Celiac artery (CA) blood flow velocities in the CA from a longitudinal abdominal section, determined close to the origin of the artery from the abdominal aorta.
4. Renal artery (RA) blood flow velocities in the right distal RA by placing the transducer in the dorsolateral area of the flank in longitudinal axis.

For the last three measurements, peak systolic velocity (PSV), end diastolic velocity (EDV), and resistive index (RI) were recorded and averaged over 3 cycles.

### Statistical method

For sample size planning, we used SPSS program version 20. A minimal total sample size of 60 (30 infants per group) is needed to study the hemodynamic and clinical differences between anemic premature infants’ group Vs. nonanemic inpatient premature infants’ group, based on Nelle et al. [16], by using Two-Sample T-Test Power Analysis in NCSS & PASS Program that detect difference of (15beats/minutes) of HR and achieves 80% power with a target significance level at 5%.

Data analysis was done using IBM SPSS software package version 20.0. The Kolmogorov-Smirnov test was used to verify the normality of distribution. Mann Whitney test and Student t-test as well as Chi-square test with Fisher’s Exact or Monte Carlo correction were used to compare the parameters in the 2 groups. Wilcoxon-signed ranks test and Paired t-test were used to compare clinical, hematological and hemodynamic parameters before and after RBCs transfusions of anemic patients. Chi-square test was used in model-I and II to compare numbers of affected hemodynamic parameters in asymptomatic-anemic, symptomatic-anemic and non-anemic-control groups. ROC-curves were used to assess the ability of different clinical and imaging parameters in the prediction of anemia along with determining different cut-off points at which sensitivity and specificity were maximized. Hemodynamic parameters in ROC curve were those that showed significant differences between anemic and non-anemic, and before and after PRBCs-transfusion. P-value of less than 0.05 was considered significant. Spearman correlation was used to measure strength of association between Hb/HCT and different hemodynamic variables.

## Results

**Fig. 1:**
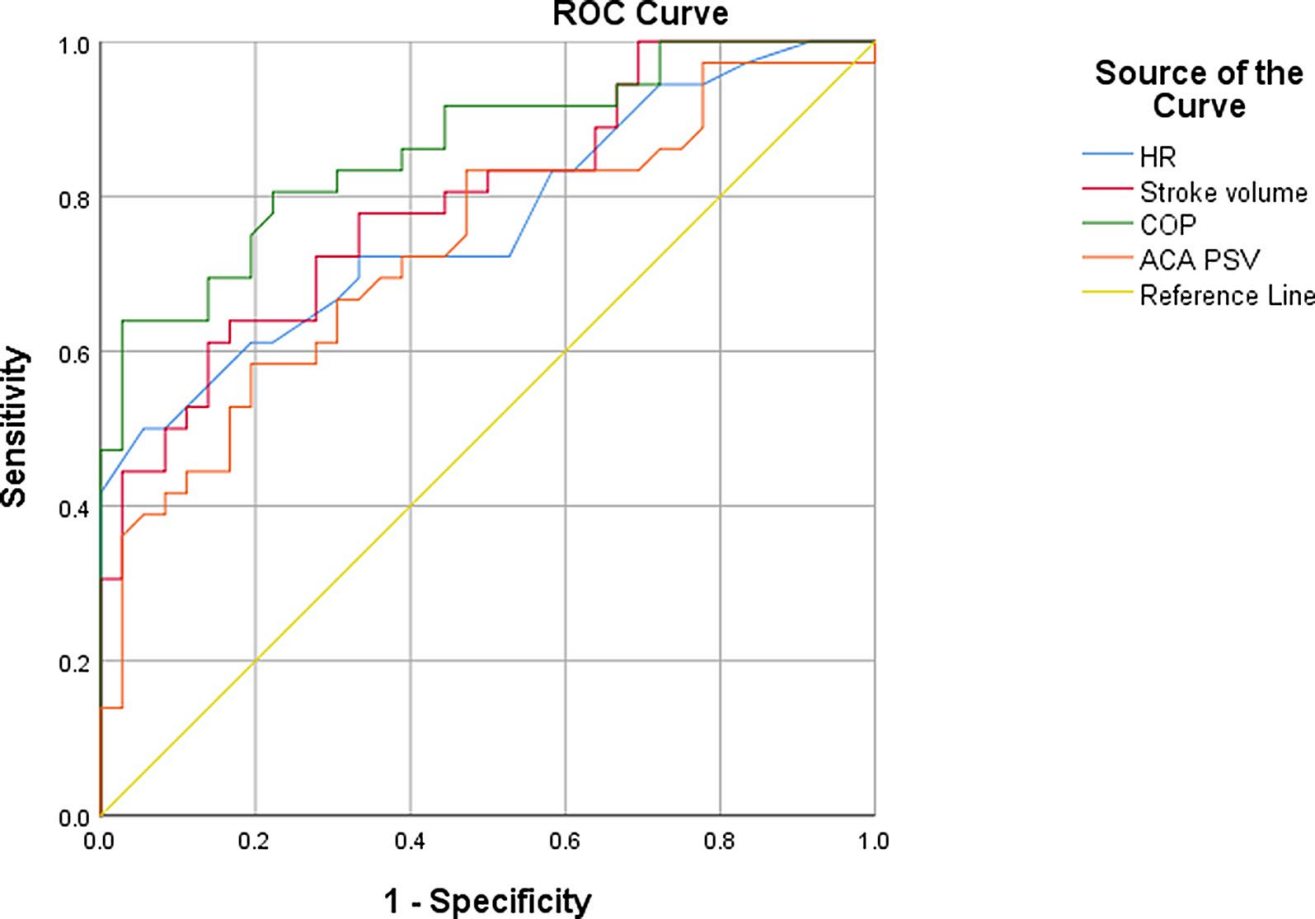
ROC curves for HR, stroke volume, COP and ACA PSV for prediction of anemia requiring PRBCs-transfusion

The study was conducted with 72 newborns with gestational age between 28 and 32 weeks and postnatal age 3–6 weeks, in order to compare clinical and imaging-based hemodynamics in anemic and non-anemic infants from one side and hemodynamic changes in anemic infants before and after RBC-transfusions from the other side, S-Figure 1.

No significant differences were seen between participants in group I and group II in terms of gestational age, gender, antenatal steroids, resuscitation needs and 1–5 min Apgar-score. Birth-weight was significantly lower and twin pregnancy was significantly higher in anemic-group. Except for maternal anemia and preterm labor pain, all maternal risk factors exhibited no significant differences between studied groups, S-Table-1.

S-Tables 2, 3 and 4 show initial blood picture of both study groups initially and at time of scan and, post-transfusion for the anemic-group. Additionally, intraventricular hemorrhage (IVH), periventricular leukomalacia (PVL) and ROP were significantly higher in anemic-group, S-Table-5.

In term of imaging based hemodynamic measures, SV, LVO and ACA-PSV were significantly higher in anemic-group, Table 1. All those hemodynamic measures significantly changed in anemic-group after having RBCs transfusions, Table 1. ACA-EDV and RA-PSV decreased significantly only after RBCs-transfusions.

In Table 2; Fig. 1, ROC was constructed to discover the cutoff values of hemodynamic parameters that were affected by anemia requiring PRBCs-transfusions. Cardiac output represented the highest diagnostic performance with area under ROC curve 0.862. A cut off value of LVO ≥ 260.2 ml /kg/min had 80.6% sensitivity and 77.8% specificity in detection of hemodynamically significant anemia. The areas under curves for SV, resting HR, and ACA-PSV were 0.793, 0.77, and 0.73, respectively.

**Table 1:** Clinical (at time of scan) and Doppler-measured hemodynamic parameters in both study group (1a). Clinical and Doppler- measured hemodynamic parameters before and after PRBCs-transfusion in the anemic group (1b)

**Table (1a)**	**Non-anemic control group** (**n = 36**)	**Anemic group** (**n = 36**)	***P***
**Postnatal age at time of examination (days)**			
Min.–Max.	21–57	21–59	0.002^*^
Mean ± SD.	25.5 ± 6.1	30.4 ± 8.6	
Median (IQR)	24 (22–27)	29.5 (23.5–33.5)	
**Hb (g/dl)**			
Min.–Max.	9.8–17	5.5–9.3	< 0.001^*^
Mean ± SD.	12.1 ± 1.4	7.4 ± 0.8	
Median (IQR)	11.9 (11.1–12.8)	7.6 (7.1–7.9)	
**HCT (%)**			
Min.–Max.	30.2–43	10–25.3	< 0.001^*^
Mean ± SD.	33.8 ± 2.9	20.6 ± 2.8	
Median (IQR)	33.3 (31.6–34.8)	21.1 (19.5–22.6)	
**SBP (mmHg)**			
Min.–Max.	60–86	52–86	0.164
Mean ± SD.	70.7 ± 5.5	68.6 ± 7.1	
Median (IQR)	70.5 (67–74)	69.5 (63–73.5)	
**DBP (mmHg)**			
Min.–Max.	40–55	27–55	0.260
Mean ± SD.	49.1 ± 4.3	47.1 ± 6.3	
Median (IQR)	50 (48–52)	49 (42–52)	
**MABP (mmHg)**			
Min.–Max.	47–66	35–66	0.286
Mean ± SD.	56.1 ± 4.9	54.2 ± 6.6	
Median (IQR)	56 (53–60)	55.5 (49–59.5)	
**Resting HR (B/min)**			
Min.–Max.	115–165	130–186	< 0.001^*^
Mean ± SD.	145.5 ± 12.6	160.4 ± 15.1	
Median (IQR)	147 (136–155)	162.5 (145–170)	
**Stroke volume (ml/kg)**			
Min.–Max.	1.03–2.42	1.34–3.33	0.017^*^
Mean ± SD.	1.56 ± 0.33	2.12 ± 0.58	
Median (IQR)	1.52 (1.32–1.82)	2.06 (1.65–2.55)	
**LVO (ml/kg/min)**			
Min.–Max.	140–322	199–570	< 0.001^*^
Mean ± SD.	226.23 ± 42.32	328.59 ± 87.01	
Median (IQR)	221.8 (190.5–258.5)	315 (263.9–377.35)	
**ACA**			
**PSV (cm/sec.)**			
Min.–Max.	26–58	22.3–76	0.001^*^
Mean ± SD.	36.92 ± 7.39	45.91 ± 12.16	
Median (IQR)	35 (33.15–40.25)	45.15 (35.9–53.15)	
**EDV (cm/sec.)**			
Min.–Max.	3.7–14	3–23	0.358
Mean ± SD.	7.09 ± 2.35	8.43 ± 4.4	
Median (IQR)	6.55 (5.6–7.65)	6.85 (5.7–10.4)	
**RI**			
Min.–Max.	0.71–0.87	0.67–0.94	0.375
Mean ± SD.	0.81 ± 0.05	0.82 ± 0.07	
Median (IQR)	0.81 (0.77–0.86)	0.82 (0.79–0.88)	
**Renal artery**			
**PSV (cm/sec.)**			
Min.–Max.	20–59	23–63	0.535
Mean ± SD.	37.63 ± 8.49	38.91 ± 8.92	
Median (IQR)	37.5 (33–42)	38.35 (33–44.5)	
**EDV (cm/sec.)**			
Min.–Max.	3.7–11.5	3–10	0.241
Mean ± SD.	6.29 ± 1.94	5.76 ± 1.64	
Median (IQR)	6.15 (5–7.15)	5.7 (4.5–6.9)	
**RI**			
Min.–Max.	0.72–0.9	0.73–0.91	0.058
Mean ± SD.	0.83 ± 0.05	0.85 ± 0.04	
Median (IQR)	0.83 (0.81–0.86)	0.86 (0.83–0.88)	
**Coeliac artery**			
**PSV (cm/sec.)**			
Min.–Max.	47–94	49–115	0.478
Mean ± SD.	67.07 ± 11.27	71.4 ± 17.45	
Median (IQR)	65 (60–73.6)	67 (59.5–81.35)	
**EDV (cm/sec.)**			
Min.–Max.	7–23.5	5.7–34.6	0.272
Mean ± SD.	14.15 ± 4.05	13.69 ± 6.24	
Median (IQR)	13.45 (11–17)	12.25 (9–16.5)	
**RI**			
Min.–Max.	0.67–0.89	0.65–0.9	0.351
Mean ± SD.	0.79 ± 0.05	0.8 ± 0.07	
Median (IQR)	0.79 (0.76–0.83)	0.81 (0.76–0.86)	
**Table (1b)**	**Before PRBCs** (**n = 36**)	**After PRBCs** (**n = 36**)	***P***
**Hb (g/dl)**			
Min.–Max.	5.5–9.3	9.0–13.0	< 0.001^*^
Mean ± SD.	7.4 ± 0.8	10.6 ± 0.9	
Median (IQR)	7.6 (7.1–7.9)	10.4 (10.0–10.9)	
**Hct (%)**			
Min.–Max.	10–25.3	26.6–39.0	< 0.001^*^
Mean ± SD.	20.6 ± 2.8	29.7 ± 2.8	
Median (IQR)	21.1 (19.5–22.6)	29.0 (28.0–30.0)	
**SBP (mmHg)**			
Min.–Max.	52–86	55.0–76.0	0.05^*^
Mean ± SD.	68.6 ± 7.1	67.3 ± 6.1	
Median (IQR)	69.5 (63–73.5)	68.0 (61.5–72.5)	
**DBP (mmHg)**			
Min.–Max.	27–55	32.0–55.0	0.061
Mean ± SD.	47.1 ± 6.3	46.3 ± 5.2	
Median (IQR)	49 (42–52)	47.5 (43.5–50.0)	
**MABP (mmHg)**			
Min.–Max.	35–66	40.0–61.0	0.119
Mean ± SD.	54.2 ± 6.6	53.2 ± 5.3	
Median (IQR)	55.5 (49–59.5)	53.5 (49.5–58.0)	
**HR (B/min)**			
Min.–Max.	130–186	130.0–185.0	< 0.001^*^
Mean ± SD.	160.4 ± 15.1	148.6 ± 12.9	
Median (IQR)	162.5 (145–170)	147.5 (140.0–158.0)	
**Stroke volume (ml/kg)**			
Min.–Max.	1.34–3.33	1.0–2.77	< 0.001^*^
Mean ± SD.	2.12 ± 0.58	1.73 ± 0.44	
Median (IQR)	2.06 (1.65–2.55)	1.64 (1.39–2.12)	
**LVO(ml/kg/min)**			
Min.–Max.	199–570	154–439.8	< 0.001^*^
Mean ± SD.	328.59 ± 87.01	257.24 ± 63.12	
Median (IQR)	315 (263.9–377.35)	245.25 (218.2–306)	
**ACA**			
**PSV (cm /Sec)**			
Min.–Max.	22.3–76	22.3–55.3	< 0.001^*^
Mean ± SD.	45.91 ± 12.16	34.11 ± 7.72	
Median (IQR)	45.15 (35.9–53.15)	32.8 (28–37.25)	
**EDV(cm /Sec)**			
Min.–Max.	3–23	3.6–12.5	0.05^*^
Mean ± SD.	8.43 ± 4.4	6.91 ± 2.14	
Median (IQR)	6.85 (5.7–10.4)	6.8 (5.25–8.45)	
**RI**			
Min.–Max.	0.67–0.94	0.7– 0.92	0.101
Mean ± SD.	0.82 ± 0.07	0.8 ± 0.05	
Median (IQR)	0.82 (0.79–0.88)	0.8 (0.78 − 0.83)	
**Renal artery (RA)**			
**PSV(cm /Sec)**			
Min.–Max.	23–63	19.5–55	< 0.001^*^
Mean ± SD.	38.91 ± 8.92	32.52 ± 7.26	
Median (IQR)	38.35 (33–44.5)	32.5 (28.5–36.5)	
**EDV(cm /Sec)**			
Min.–Max.	3–10	3–11	0.702
Mean ± SD.	5.76 ± 1.64	5.84 ± 1.67	
Median (IQR)	5.7 (4.5–6.9)	5.6 (5–7)	
**RI**			
Min.–Max.	0.73–0.91	0.7–0.91	0.011^*^
Mean ± SD.	0.85 ± 0.04	0.82 ± 0.05	
Median (IQR)	0.86 (0.83–0.88)	0.83 (0.79–0.85)	
**Coeliac artery (CA)**			
**PSV(cm /Sec)**			
Min.–Max.	49–115	44–112	0.122
Mean ± SD.	71.4 ± 17.45	66.42 ± 15.59	
Median (IQR)	67 (59.5–81.35)	66.35 (57.5–72.5)	
**EDV(cm /Sec)**			
Min.–Max.	5.7–34.6	6–22	0.330
Mean ± SD.	13.69 ± 6.24	12.28 ± 3.93	
Median (IQR)	12.25 (9–16.5)	12 (10–14.5)	
**RI**			
Min.–Max.	0.65–0.9	0.61–0.92	0.609
Mean ± SD.	0.8 ± 0.07	0.81 ± 0.06	
Median (IQR)	0.81 (0.76–0.86)	0.82 (0.79–0.86)	

SD: Standard deviation, Z: Wilcoxon signed ranks test, t: Paired t-test

U: Mann Whitney test, t: Student t-test

p: p value for comparing between two groups

*: Statistically significant at *p* ≤ 0.05

LVO: left ventricular output, ACA: Anterior cerebral artery, PSV: Peak systolic velocity

EDV: End diastolic velocity, RI: Resistance index

LVO, a measure of systemic blood flow is a product of stroke volume (SV) and HR. As a compensatory mechanism to increase the oxygenation of brain parenchyma, the LVO rises in anemia. Increased LVO may be due to increased contractility, HR, and flow-velocities. The patho-physiological mechanisms behind high cardiac output might be increased hypoxia-stimulated chemoreceptors, lower blood viscosity, hypoxia-induced vasodilatation (decreased afterload), enhanced nitic oxide activity, and increased catecholamine and non-catecholamine inotropic factors [26]. The majority of anemic patients had high LVO, 19/24 of anemic symptomatic patients and 9/12 of anemic asymptomatic patients. LVO has AUC in diagnosis of anemia 0.86, Fig. 1; Table 2. It is interesting to note that part of patients with high LVO do not have a resting heart rate greater than 151beats per minute nor SV greater than 1.69 ml /kg/beat. This might indicate that LVO can detect early changes in hemodynamics caused by anemia when the SV and HR are not yet significantly increased. This coincides with what has been reported for a long time, that an increase in cardiac output is the first physiological reaction to anemia [27]. 

**Table 2:** The predictive values of HR, stroke volume, COP and ACA PSV for hemodynamically significant anemia of prematurity

	*P*	AUC	95% C.I	Cut off	Sensitivity	Specificity	PPV	NPV
HR	< 0.001^*^	0.767	0.658–0.876	≥ 151b/min	72.2	66.7	68.4	70.6
Stroke volume	< 0.001^*^	0.793	0.691–0.895	≥ 1.69 ml/kg	72.2	72.2	72.2	72.2
LVO	< 0.001^*^	0.862	0.778–0.946	≥ 260.2 ml/kg/min	80.6	77.8	78.4	80
ACA PSV	0.001^*^	0.732	0.615–0.848	≥ 38.65 cm/sec	66.7	69.4	68.6	67.6

**Fig. 2:**
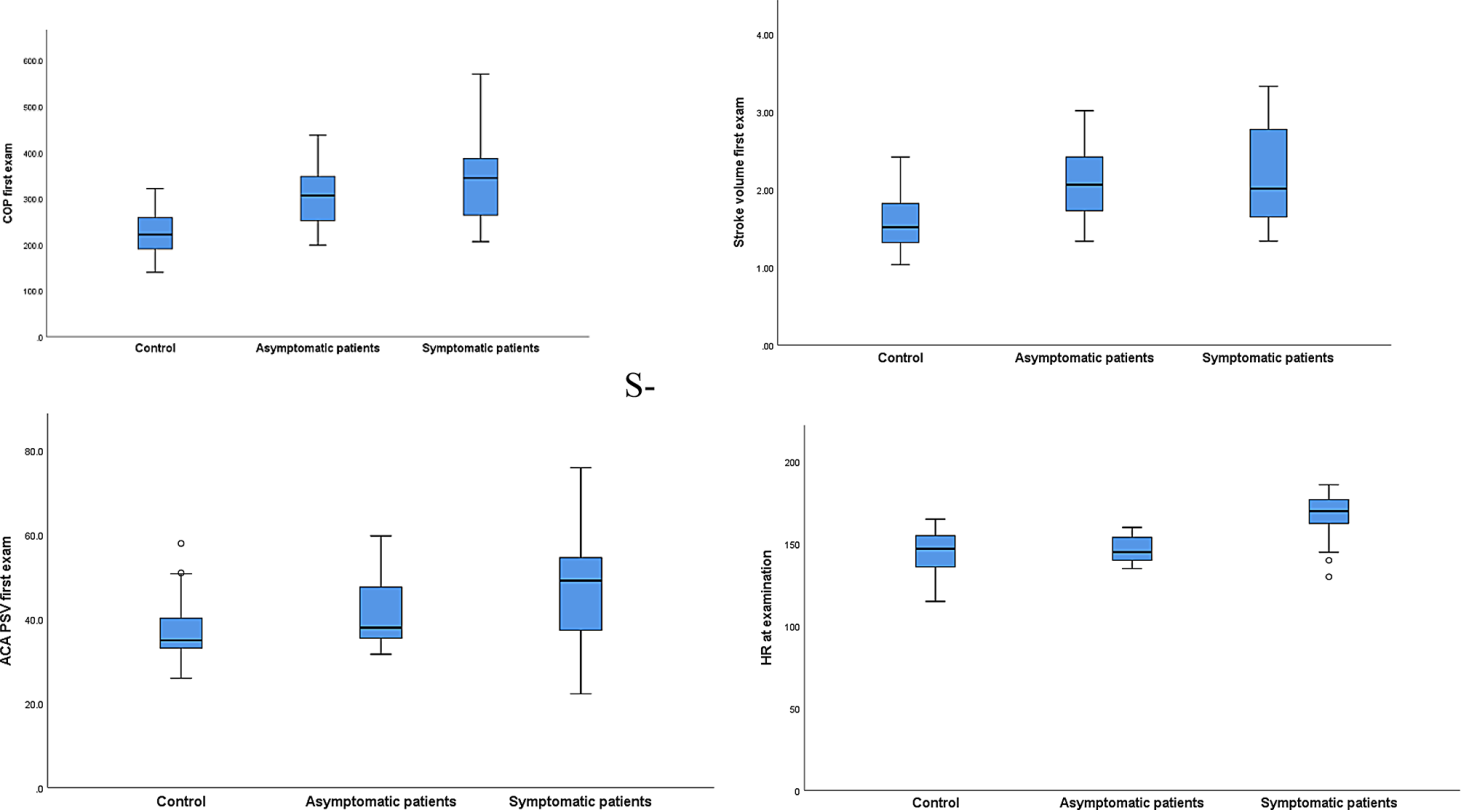
Box blot graphs for resting HR, SV, LVO and ACA-PSV in the three groups (nonanemic-control, asymptomatic-anemic and asymptomatic-anemic groups)

**Fig. 3:**
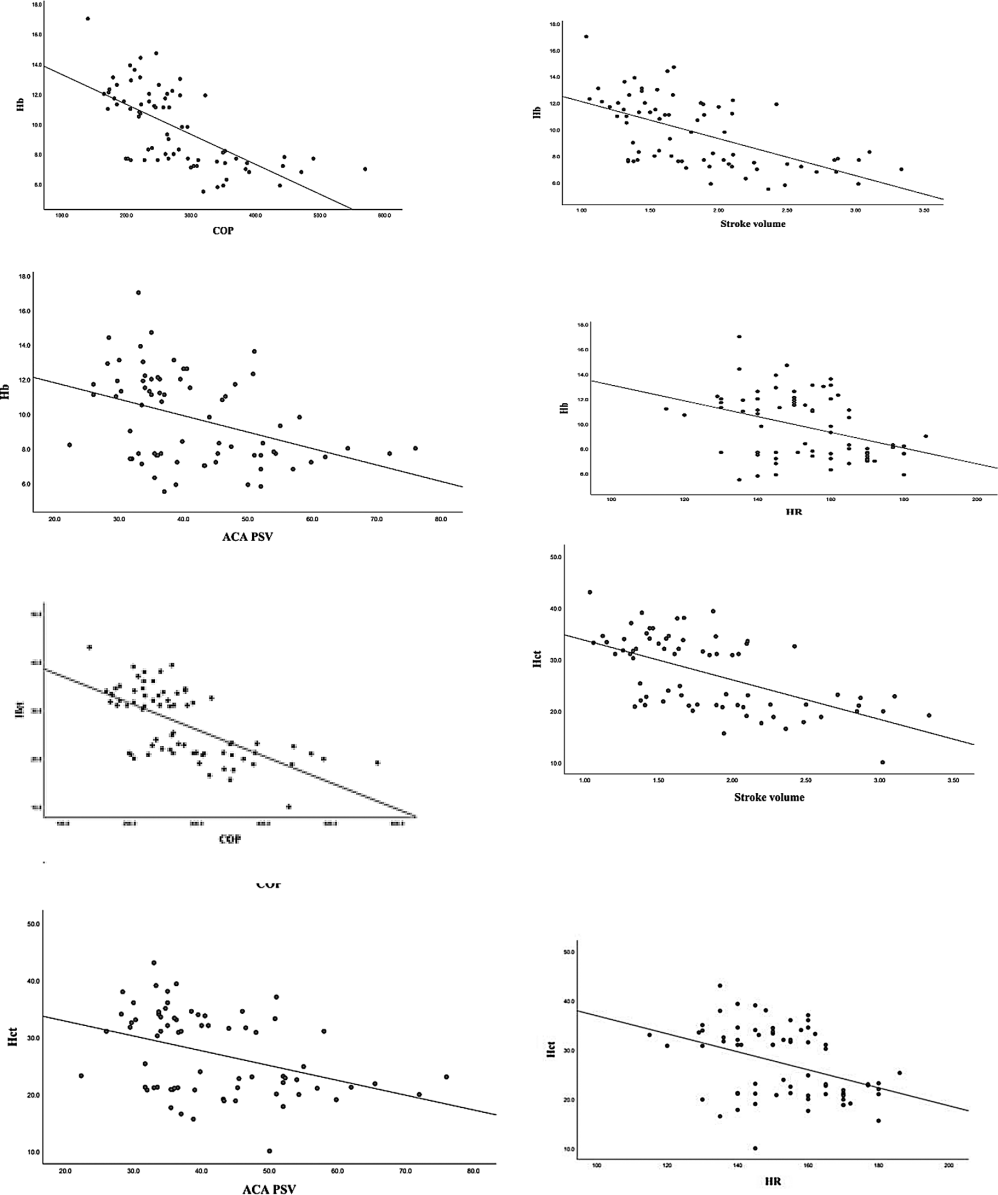
Correlation between Hb/Hct and different hemodynamic parameters. Upper panel shows correlation between HB and different hemodynamic parameters (lvo, SV, ACA-PSV, and HR) with rho − 0.683(*p* = 0.001), -0.598(*p* = 0.001), -0.389(*p* = 0.001), and − 0.345(*p* = 0.003), respectively. Lower panel shows correlation between HB and different hemodynamic parameters (LVO, SV, ACA-PSV, and HR) with rho − 0.635 (*p* = 0.001), -0.559 (*p* = 0.001), -0.389(*p* = 0.001), and − 0.359 (*p* = 0.002), respectively

Table 3, anemic patients were further subdivided into anemic-symptomatic (*n* = 24) and anemic-asymptomatic (*n* = 12) patients. Hemodynamic parameters using ROC-curves’ cut off values showed statistically significant differences among the three groups in the 2 models. Figure 2, hemodynamic parameters were demonstrated in non-anemic, asymptomatic-anemic, and symptomatic- anemic patients. Figure 3 showed significant correlation between Hb/HCT and different hemodynamic parameters (LVO, SV, ACA-PSV, and HR) with almost similar correlation coefficient.

**Table 3:** Comparison between the three groups (non-anemic, asymptomatic and symptomatic anemic patients) regarding number of affected hemodynamic parameters using cutoff values of ROC curves

		**Cases (anaemic)**				
		**Control(non-anemic)**	**Asymptomatic Patients**	**Symptomatic Patients**		
Affected Parameters	0	11	0	0		
	1	12	2	1		
	2	11	4	3		
	3	1	5	11		
	4	1	1	9		
		**Cases (anaemic)**			**χ** ^**2**^ **test**	**P**
		**Control (nonanemic)**	**Asymptomatic Patients**	**Symptomatic Patients**		
Model-I: Affected Parameters	≤ 1	23 ^a^	2^b^	1^b^	24.622	< 0.001^*^
		63.9%	16.7%	4.2%		
	> 1	13^a^	10^b^	23^b^		
		36.1%	83.3%	95.8%		
Model-II: Affected Parameters	≤ 2	34 ^a^	6^b^	4^b^	37.403	< 0.001^*^
		94.4%	50.0%	16.7%		
	> 2	2^a^	6^b^	20^b^		
		5.6%	50.0%	83.3%		

χ2: Chi-square test

p: p-value for comparing between the three studied groups

*: Statistically significant at *p* ≤ 0.05

In each row: different letters are significant

## Discussion

The assessment of hemodynamic parameters by neonatologists enables the delivery of care that is both physiology-based and disease-specific for preterm infants suffering from various complications related to prematurity [17–21]. In our research, we explored the hemodynamic impact of anemia and the alterations in hemodynamics following RBC transfusions in anemic preterm infants. Hemoglobin (Hb) and hematocrit (HCT) alone do not sufficiently indicate tissue oxygenation or the necessity for transfusion [9, 11]. The oxygen delivery before and after transfusion, as well as in both anemic and non-anemic neonates, may not differ significantly due to compensatory increases in heart rate (HR) and stroke volume (SV) for low Hb levels. However, this compensation comes with energy costs. Our findings align with previous studies that also examined hemodynamic changes in anemia or post-PRBC transfusion in neonates [22–25]. Furthermore, the potential inclusion of hemodynamic markers as objective criteria in blood transfusion decisions was previously proposed by Quante et al. [24] In our study, we analyzed various hemodynamic parameters in anemic patients before and after transfusion and compared them with those of a non-anemic control group. Additionally, we evaluated different hemodynamic measures in asymptomatic and symptomatic anemic patients to assess the reliability of these clinical signs in deciding on PRBC transfusion. Left ventricular output (LVO) demonstrated the highest performance in the ROC curve and the strongest correlation with Hb/HCT, followed by SV, HR, and ACA-PSV.

We found that LVO, SV, HR and ACA-PSV were significantly higher not only in anemic-group in comparison with the control group, but also in anemic group before and after RBCs-transfusions. Therefore, we constructed ROC-curves for those variables to determine the cut off points that can help in taking the decision of RBCs-transfusions in anemic premature infants at postnatal age of 3–9 weeks. Then, we classified patients into control (group I), asymptomatic-anemic and symptomatic-anemic patients in two models (model-I and-II in Table 3). We found that there were significant differences among the three groups regarding number of affected hemodynamic parameters, according to the cut off points of ROC-curves. Recently, NIRS-measured regional oxygenation has been introduced as marker of tissue oxygenation that can be used to decide transfusion needs in preterm infants. However, high expenses of the sensors might be unpractical for every patient use in developing countries. In the current work we used echocardiography and Doppler as they are inexpensive imaging tools and can be easily obtained in limited resource areas, compared to NIRS. They are practical tools that can be used by the clinicians in combination with HCT/HB levels and clinical signs of anemia to decide which and when patients are truly candidates for RBCs-transfusion.

Normovolemic anemia may lead to cerebral hyperemia as a compensatory mechanism, resulting in increased cerebral blood flow (CBF) due to reduced blood viscosity, compensatory cerebral vasodilation, and elevated cardiac output [28]. In anemic patients, ACA-PSV is notably higher compared to those without anemia.

Immature splanchnic tissues could be at lower risk of hypoxia than cerebral and renal tissues in anemic preterm infants because of lower baseline oxygenation (which probably indicates a lower oxygen requirement) and the higher oxygen extraction ability [29]. In other words, intestinal oxygenation in those age group depends on an enhanced ability to extract oxygen from the blood rather than change in blood flow [30]. This might explain why CA- velocities were not affected neither by anemia or RBCs-transfusions.

In the current study asymptomatic anemic patients showed no clinical signs or increased oxygen requirements despite of reaching critically low level of haemoglobin. Also, as shown in Table 3, there were no statistically significant difference as regards number of hemodynamic parameters affected between symptomatic and asymptomatic anemic patients, while the true statistically difference was found between non-anemic controls and both anemic symptomatic and asymptomatic patients. This indicates that depending on clinical data might be fallacious and it might be the time to replace or at least combine the clinical data with imaging-based hemodynamic measures and Hb/HCT.

As shown in model 2 (Table  3), 34 patients in non-anemic-group have 2 or fewer parameters affected, while only 2 patients have three parameters affected. Interestingly, the Hb values of those affected patients are the lowest of the entire control group, 9.8 g/dl for both while HCT values were above 30. Both patients had LVO > 262 ml/kg/min. One of these two patients developed stage III-ROP and NEC at DOL25. This may suggest that those patients were in need of PRBCs-transfusions. This may lead us to clinical conclusion that Hb is better than HCT for monitoring anemia [31, 32]. In the case of large RBCs, the HCT will be falsely high. Patients’ volume status affects both indices, but it has a less significant impact on HB than HCT. Figure 3 indicates that HCT and HB might be interchangeable in their effect on hemodynamics.

In model-I, every patient experiencing symptomatic anemia has at least one parameter that is affected. These hemodynamic measurements could potentially minimize the subjectivity of clinical data in patients with symptomatic anemia and assist clinicians in determining the necessity of RBC transfusions. Patients with asymptomatic anemia who do not exhibit altered hemodynamic measures might not require RBC transfusions at that time, according to model-I and model-II. As shown in Table 3, only one patient in the symptomatic anemic group had just one affected parameter, which was the resting heart rate, while the other patients had at least two affected parameters. Notably, this patient’s heart rate remained unchanged before and after the transfusion. It is probable that this patient did not have hemodynamically significant anemia and would not need PRBC transfusion, with the tachycardia likely being caused by another factor. PVL, ROP, and BPD share nearly identical pathologies, which are attributed to anemia-induced hypoxia and oxidative damage from RBC transfusions. This is due to an increase in non-transferrin bound iron or inflammatory mediators found in stored blood products [3–5]. In this study, there were statistically significant differences between the two groups concerning PVL and ROP. However, no statistically significant differences were observed between the groups regarding BPD, even though the anemic group had four cases of BPD, while the non-anemic group had none. BPD is characterized by a complex and multifactorial pathology, and invasive mechanical ventilation, a major risk factor, was similarly prevalent in both groups. (S-Table 5).

## Limitations

In the current work, we studied the hemodynamic changes with the current cut off values of anemia, while the cut off values based on both Hb and hemodynamic parameters require more validation by long term follow up of patients with randomized controlled trials.

Long term follows up of those patients was not feasible in this work. No modification of the management plan based on measures as the cutoff values were not known before analyzing data at the end of studies. Further limitation is relatively small sample size and being from one Centre, however, we introduced the idea of incorporation of hemodynamic measures in decision of RBCs-transfusion.

The recommendation to add parameters obtained from noninvasive Doppler imaging and functional echocardiography evaluation might be available techniques that are less costly and more generally applicable. However, there is still the issue of educating medical workers on Doppler imaging and echocardiography as well as subjectivity and reproducibility in ultrasound examinations.

In addition, hemodynamic measurements cannot predict or even relate to anemia symptoms. This means that some patients remained clinically quiet while being hemodynamically affected. Similarly, the appearance of anemic symptoms does not always imply that more hemodynamic parameters are affected.

The hemodynamic changes in anemic neonates have developed gradually over days and corrected in few hours. Thus, there is also a question to be debated after having significant changes in hemodynamics following PRBC transfusions in anemic patients: can rapid changes in hemodynamics harm those premature patients? This question remains unsolved in the current study.

## **Conclusion**s

In clinical settings, hemodynamic measurements are being utilized more frequently. For anemic premature infants with a gestational age of 32 weeks or less and a postnatal age ranging from 3 to 9 weeks, specific thresholds such as LVO ≥ 260.2 ml/kg/min, ACA-PSV ≥ 38.65 cm/sec, SV ≥ 1.69 ml/kg/beat, and a resting heart rate of ≥ 151 beats/min can assist clinicians in customizing transfusion thresholds for each patient, thereby improving the accuracy of PRBCs transfusion decisions. Additionally, the involvement of more than two variables may indicate HS-AOP in this age group. Further research is necessary to confirm these cut-off values. Both Hb and HCT can be used interchangeably to identify HS-AOP.

## Electronic supplementary material

Below is the link to the electronic supplementary material.

Supplementary Material 1

## Data Availability

The datasets used and/or analysed during the current study are available from the corresponding author on reasonable request.

## Abbreviations

ACA: Anterior cerebral artery
AOP: Anemia of prematurity
BPD: Bronco-pulmonary dysplasia
BP: Blood pressure
CBF: Cerebral blood flow
CA: Celiac artery
EDV: End diastolic velocity
GA: Gestational age
RBCs: Red blood cells
Hb: Hemoglobin
HCT: Hematocrit
HS: Hemodynamically significant
HR: Heart rate
IVH: Intraventricular hemorrhage
LVO: Left ventricular cardiac output
NEC: Necrotizing enterocolitis
NICU: Neonatal intensive care
PRBCs: Packed red blood cells
PSV: Peak systolic velocity
PVL: Periventricular hemorrhage
ROP: Retinopathy of prematurity
RA: Renal artery
SV: Stroke volume
VTI: Velocity time integral
