# Supplementary material for: Hemodynamically significant anemia as an indication of transfusion in preterm infants
Source: Ital J Pediatr. 2025 May 16;51:140. doi: 10.1186/s13052-025-01978-w (PMC12084977; doi:10.1186/s13052-025-01978-w)
Supplement: Supplementary file 1 — Supplementary Material 1 [file 13052_2025_1978_MOESM1_ESM.docx]

**S-Figure 1: Flow chart of the study**

**S-Table (1): Socioeconomic and demographic data of both study groups.**

|  | | | **Control (group I)**  **(n=36)** | **Cases (groupII)**  **(n=36)** | **Test of sig.** | **P** |
| --- | --- | --- | --- | --- | --- | --- |
| **Sex** | Male | | 15 (41.7%) | 18 (50.0%) | χ^2^= 0.503 | 0.478 |
|  | Female | | 21 (58.3%) | 18 (50.0%) |  |  |
| **GA (weeks)** | | |  |  |  |  |
| Min. – Max. | | | 29.0 – 32.0 | 28.0 – 32.0 | U= 561 | 0.305 |
| Mean ± SD. | | | 31.0 ± 1.0 | 30.6 ± 1.2 |  |  |
| Median (IQR) | | | 31.0 (30.0 – 32.0) | 31.0 (30.0 – 32.0) |  |  |
| **Birth weight (kg)** | | |  |  |  |  |
| Min. – Max. | | | 0.8 – 1.5 | 0.7 – 1.4 | t= 3.949 | <0.001**^*^** |
| Mean ± SD. | | | 1.2 ± 0.2 | 1.1 ± 0.2 |  |  |
| Median (IQR) | | | 1.2 (1.1 – 1.3) | 1.0 (0.9 – 1.2) |  |  |
| **Fertilization method** | Spontaneous | | 34 (94.4%) | 30 (83.3%) | χ^2^= 4.821 | ^MC^p = 0.107 |
|  | ICSI | | 1 (2.8%) | 6 (16.7%) |  |  |
|  | Ovulation induction | | 1 (2.8%) | 0 (0%) |  |  |
| **Mode of delivery** | CS | | 28 (77.8%) | 28 (77.8%) | χ^2^= 0.00 | 1.000 |
|  | NVD | | 8 (22.2%) | 8 (22.2%) |  |  |
| **Multiplicity** | Single | | 26 ^a^ (72.2%) | 17 ^b^ (47.2%) | χ^2^= 6.884 | ^MC^p = 0.038**^*^** |
|  | Twin | | 9 ^a^ (25%) | 18 ^b^ (50%) |  |  |
|  | Triplet | | 1 ^a^ (2.8%) | 0 ^a^ (0%) |  |  |
|  | Quadriplet | | 0 ^a^ (0%) | 1 ^a^ (2.8%) |  |  |
| **Parity** | Primipara | | 12 (33.3%) | 15 (41.7%) | χ^2^= 0.533 | 0.465 |
|  | Multipara | | 24 (66.7%) | 21 (58.3%) |  |  |
| **Antenatal care** | No | | 5 (13.9%) | 3 (8.3%) | χ^2^= 0.563 | FEp = 0.710 |
|  | Yes | | 31 (86.1%) | 33 (91.7%) |  |  |
| **Antenatal steroids** | No | | 11 (30.6%) | 9 (25%) | χ^2^= 2.200 | 0.333 |
|  | Incomplete | | 10 (27.8%) | 16 (44.4%) |  |  |
|  | Complete | | 15 (41.7%) | 11 (30.6%) |  |  |
| **Resuscitation** | Initial steps | | 27 (75%) | 29 (80.6%) | χ^2^= 0.321 | 0.571 |
|  | PPV | | 9 (25%) | 7 (19.4%) |  |  |
| **APGAR 1 min** | | |  |  |  |  |
| Min. – Max. | | | 2 – 7 | 3 – 7 | U= 557 | 0.272 |
| Mean ± SD. | | | 5.8 ± 1.2 | 5.6 ± 1.1 |  |  |
| Median (IQR) | | | 6 (5.5 – 7) | 6 (5 – 6) |  |  |
| **APGAR 5 min** | | |  |  |  |  |
| Min. – Max. | | | 5 – 9 | 7 – 9 | U= 595 | 0.493 |
| Mean ± SD. | | | 8 ± 0.7 | 7.9 ± 0.7 |  |  |
| Median (IQR) | | | 8 (8 – 8) | 8 (7 – 8) |  |  |
| **Maternal anaemia** | | No | 25 (69.4%) | 14 (38.9%) | χ^2^= 6.769 | 0.009**^*^** |
|  |  | Yes | 11 (30.6%) | 22 (61.1%) |  |  |
| **Preeclampsia** | | No | 24 (66.7%) | 28 (77.8%) | χ^2^= 1.108 | 0.293 |
|  |  | Yes | 12 (33.3%) | 8 (22.2%) |  |  |
| **Accidental haemorrhage** | | No | 32 (88.9%) | 29 (80.6%) | χ^2^= 0.966 | 0.326 |
|  |  | Yes | 4 (11.1%) | 7 (19.4%) |  |  |
| **PTLP** | | No | 20 (55.6%) | 11 (30.6%) | χ^2^= 4.589 | 0.032**^*^** |
|  |  | Yes | 16 (44.4%) | 25 (69.4%) |  |  |
| **Vaginitis** | | No | 28 (77.8%) | 21 (58.3%) | χ^2^= 3.130 | 0.077 |
|  |  | Yes | 8 (22.2%) | 15 (41.7%) |  |  |
| **UTI** | | No | 27 (75%) | 24 (66.7%) | χ^2^= 0.605 | 0.437 |
|  |  | Yes | 9 (25%) | 12 (33.3%) |  |  |
| **DM** | | No | 35 (97.2%) | 32 (88.9%) | χ^2^= 1.934 | FEp = 0.357 |
|  |  | Yes | 1 (2.8%) | 4 (11.1%) |  |  |
| **PROM** | | No | 29 (80.6%) | 24 (66.7%) | χ^2^= 1.787 | 0.181 |
|  |  | Yes | 7 (19.4%) | 12 (33.3%) |  |  |

χ2: Chi-square test FE: Fisher Exact U: Mann Whitney test

p: p-value for comparing between the three studied groups *: Statistically significant at p ≤ 0.05

UTI urinary tract infection PTLP preterm labour pain DM diabetes mellitus PROM premature rupture of membrane

**S-Table (2): Comparison between the two studied groups as regards initial blood picture**

|  | **Non-anemic group**  **(n=36)** | **Anemic group**  **(n=36)** | **Test of sig.** | **P** |
| --- | --- | --- | --- | --- |
| **Hb(gm/dl)** |  |  |  |  |
| Min. – Max. | 14.2 – 20.7 | 10.8 – 18.7 | t= 5.881 | <0.001**^*^** |
| Mean ± SD. | 17.1 ± 1.7 | 14.5 ± 2.1 |  |  |
| Median (IQR) | 17 (16 – 18.2) | 14.4 (12.8 – 15.7) |  |  |
| **HCT%** |  |  |  |  |
| Min. – Max. | 40 – 60 | 28.6 – 51 | t= 6.211 | <0.001**^*^** |
| Mean ± SD. | 48.1 ± 4.8 | 40.5 ± 5.7 |  |  |
| Median (IQR) | 47.7 (44 – 50.5) | 41.9 (36.5 – 44.6) |  |  |
| **MCV (fl)** |  |  |  |  |
| Min. – Max. | 85 – 119 | 79.8 – 128 | U= 593 | 0.535 |
| Mean ± SD. | 107.5 ± 7.7 | 108.5 ± 11.2 |  |  |
| Median (IQR) | 109 (105 – 111) | 109.5 (104.5 – 115) |  |  |
| **MCH (pg)** |  |  |  |  |
| Min. – Max. | 30 – 44 | 27.2 – 52.5 | U= 641.5 | 0.942 |
| Mean ± SD. | 38.2 ± 3 | 38.4 ± 5.2 |  |  |
| Median (IQR) | 38 (36.2 – 40.1) | 38 (36 – 41) |  |  |
| **WBCs (10^3^/ul)** |  |  |  |  |
| Min. – Max. | 3.1 – 105 | 4.7 – 30 | U= 590 | 0.513 |
| Mean ± SD. | 12.4 ± 16.5 | 11.7 ± 6.5 |  |  |
| Median (IQR) | 10 (6.4 – 12) | 9.9 (7.3 – 14.6) |  |  |
| **PLT(10^3^/ul)** |  |  |  |  |
| Min. – Max. | 15 – 334 | 67 – 504 | U= 624 | 0.787 |
| Mean ± SD. | 192.8 ± 75 | 206.2 ± 91.1 |  |  |
| Median (IQR) | 211 (140 – 243.5) | 199 (142.5 – 245.5) |  |  |

U: Mann Whitney test t: Student t-test

p: p-value for comparing between the **two studied groups**

**S-Table (**3**): Comparison between the two studied groups as regards blood picture at the time of exam**

|  | **Non-anemic group**  **(n=36)** | **Anaemic group**  **(n=36)** | **Test of sig.** | **P** |
| --- | --- | --- | --- | --- |
| **Hb (gm/dl)** |  |  |  |  |
| Min. – Max. | 9.8 – 17 | 5.5 – 9.3 | U= 0 | <0.001**^*^** |
| Mean ± SD. | 12.1 ± 1.4 | 7.4 ± 0.8 |  |  |
| Median (IQR) | 11.9 (11.1 – 12.8) | 7.6 (7.1 – 7.9) |  |  |
| **Hct (%)** |  |  |  |  |
| Min. – Max. | 30.2 – 43 | 10 – 25.3 | U= 0 | <0.001**^*^** |
| Mean ± SD. | 33.8 ± 2.9 | 20.6 ± 2.8 |  |  |
| Median (IQR) | 33.3 (31.6 – 34.8) | 21.1 (19.5 – 22.6) |  |  |
| **MCV (fl)** |  |  |  |  |
| Min. – Max. | 75 – 110 | 71.6 – 117.5 | t= 1.290 | 0.201 |
| Mean ± SD. | 94 ± 7.6 | 91.5 ± 8.7 |  |  |
| Median (IQR) | 93.5 (90.3 – 98.5) | 91.1 (87.5 – 96.6) |  |  |
| **MCH (pg)** |  |  |  |  |
| Min. – Max. | 25 – 40 | 26 – 45.7 | t= 1.618 | 0.110 |
| Mean ± SD. | 34.4 ± 3.1 | 33.1 ± 3.7 |  |  |
| Median (IQR) | 34.5 (32.7 – 36.9) | 33 (31 – 34.9) |  |  |
| **WBC (10^3^/ul)** |  |  |  |  |
| Min. – Max. | 5.6 – 24 | 4.7 – 21 | U= 465.5 | 0.04**^*^** |
| Mean ± SD. | 11.2 ± 3.7 | 9.9 ± 4.1 |  |  |
| Median (IQR) | 10.4 (9.5 – 12.4) | 8.4 (7 – 12) |  |  |
| **PLT (10^3^/ul)** |  |  |  |  |
| Min. – Max. | 134 – 632 | 140 – 874 | U= 525 | 0.166 |
| Mean ± SD. | 364.8 ± 124.5 | 430.1 ± 178.1 |  |  |
| Median (IQR) | 347.5 (267.5 – 442.5) | 400 (295.5 – 536.5) |  |  |

U: Mann Whitney test t: Student t-test p: p-value for comparing between the **two studied groups**

**S-Table (4): Comparison between before and after PRBCs transfusion in the cases group as regards blood picture at the time of exam (36)**

|  | **Before PRBCs**  **(n=36)** | **After PRBCs**  **(n=36)** | **Test of sig.** | **P** |
| --- | --- | --- | --- | --- |
| **Hb (g/gl)** |  |  |  |  |
| Min. – Max. | 5.5 – 9.3 | 9.0 – 13.0 | Z= -5.233 | <0.001**^*^** |
| Mean ± SD. | 7.4 ± 0.8 | 10.6 ± 0.9 |  |  |
| Median (IQR) | 7.6 (7.1 – 7.9) | 10.4 (10.0 – 10.9) |  |  |
| **Hct (%)** |  |  |  |  |
| Min. – Max. | 10 – 25.3 | 26.6 – 39.0 | Z= -5.232 | <0.001**^*^** |
| Mean ± SD. | 20.6 ± 2.8 | 29.7 ± 2.8 |  |  |
| Median (IQR) | 21.1 (19.5 – 22.6) | 29.0 (28.0 – 30.0) |  |  |
| **MCV (fl)** |  |  |  |  |
| Min. – Max. | 71.6 – 117.5 | 47.4 – 100.3 | Z= -2.074 | 0.038**^*^** |
| Mean ± SD. | 91.5 ± 8.7 | 87.3 ± 9.5 |  |  |
| Median (IQR) | 91.1 (87.5 – 96.6) | 89.5 (84.0 – 92.8) |  |  |
| **MCH (pg)** |  |  |  |  |
| Min. – Max. | 26 – 45.7 | 26.0 – 37.0 | t= 2.087 | 0.044**^*^** |
| Mean ± SD. | 33.1 ± 3.7 | 31.8 ± 2.8 |  |  |
| Median (IQR) | 33 (31 – 34.9) | 31.9 (30.0 – 34.0) |  |  |
| **WBC(10^3^/ul)** |  |  |  |  |
| Min. – Max. | 4.7 – 21 | 5.0 – 18.0 | Z= -0.094 | 0.925 |
| Mean ± SD. | 9.9 ± 4.1 | 9.4 ± 2.6 |  |  |
| Median (IQR) | 8.4 (7 – 12) | 9.2 (7.9 – 10.9) |  |  |
| **PLT (10^3^/ul)** |  |  |  |  |
| Min. – Max. | 140 – 874 | 136.0 – 685.0 | Z= -2.924 | 0.003**^*^** |
| Mean ± SD. | 430.1 ± 178.1 | 366.4 ± 124.1 |  |  |
| Median (IQR) | 400 (295.5 – 536.5) | 329.5 (288.5 – 431.5) |  |  |

SD: **Standard deviation Z: Wilcoxon signed ranks test t: Paired t-test**

p: p value for comparing between two groups

***: Statistically significant at p ≤ 0.05**

**S-Table (5): prematurity related complications in the two study groups**

|  | | **Control**  **(n=36)** | **Cases**  **(n=36)** | **Test of sig.** | **P** |
| --- | --- | --- | --- | --- | --- |
| **IVH** | No | 28 (77.8%) | 11 (30.6%) | χ^2^= 16.168 | <0.001**^*^** |
|  | Yes | 8 (22.2%) | 25 (69.4%) |  |  |
| **PVL** | No | 26 (72.2%) | 12 (33.3%) | χ^2^= 10.923 | 0.001**^*^** |
|  | Yes | 10 (27.8%) | 24 (66.7%) |  |  |
| **BPD** | No | 36 (100%) | 32 (88.9%) | χ^2^= 4.235 | FEp = 0.115 |
|  | Yes | 0 (0%) | 4 (11.1%) |  |  |
| **ROP** | No | 33 (91.7%) | 24 (66.7%) | χ^2^= 6.821 | 0.009**^*^** |
|  | Yes | 3 (8.3%) | 12 (33.3%) |  |  |
| **NEC≥ stage II** | No | 35 (97.2%) | 34 (94.4%) | χ^2^= 0.348 | FEp = 1.000 |
|  | Yes | 1 (2.8%) | 2 (5.6%) |  |  |
| **Duration of hospital stay** |  |  |  | U=291.5 | <0.001**^*^** |
| Min. – Max. |  | 24 – 90 | 25 – 85 |  |  |
| Mean ± SD. |  | 37.1 ± 11.4 | 49.4 ± 14.2 |  |  |
| Median(IQR) |  | 35 (30.5 – 42) | 50 (40 – 61.5) |  |  |
| **Fate** discharged |  | 36 (100%) | 32 (88.9%) | χ^2^= 4.235 | FEp = 0.115 |
| death |  | 0 (0%) | 4 (11.1%) |  |  |
| **Need for IMV** |  |  |  | χ^2^= 2.667 | 0.102 |
| yes |  | 30 (83.3%) | 24 (66.7%) |  |  |
| No |  | 6 (16.7%) | 12 (33.3%) |  |  |
| **Duration of** | **IMV** |  |  | U= 24.5 | 0.258 |
| Min. – Max.  Mean ± SD.  Median(IQR) |  | 1 – 4 | 3 – 8 |  |  |
|  |  | 3.2 ± 1.2 | 4.7 ± 2 |  |  |
|  |  | 3.5 (3 – 4) | 4 (3 – 6.5) |  |  |
| **Duration of NCPAP in days (n=69)**  Min. – Max.  Mean ± SD.  Median (IQR) | |  |  | U= 406 | 0.021**^*^** |
|  |  | 1 – 7 | 1 – 30 |  |  |
|  |  | 2.5 ± 1.4 | 4.8 ± 5.5 |  |  |
|  |  | 2 (2 – 3) | 3 (2 – 5.5) |  |  |

χ2: Chi-square test FE: Fisher Exact

p: p-value for comparing between the three studied groups *: Statistically significant at p ≤ 0.05

IVH: Intraventricular haemorrhage PVL: Periventricular leukomalacia BPD: Bronchopulmonary dysplasia

ROP: Retinopathy of prematurity NEC: Necrotizing enterocolitis IMV invasive mechanical ventilation

U: Mann Whitney test t: Student t-test

SD: Standard deviation Z: Wilcoxon signed ranks test t: Paired t-test
